# Effects and prediction of cognitive load on encoding model of brain response to auditory and linguistic stimuli in educational multimedia

**DOI:** 10.1038/s41598-024-59411-x

**Published:** 2024-04-21

**Authors:** Amir Hosein Asaadi, S. Hamid Amiri, Alireza Bosaghzadeh, Reza Ebrahimpour

**Affiliations:** 1 Department of Computer Engineering, Shahid Rajaee Teacher Training University, Tehran, Islamic Republic of Iran; 2 Institute for Research in Fundamental Sciences (IPM), School of Cognitive Sciences, Tehran, Iran; 3 Center for Cognitive Science, Institute for Convergence Science and Technology (ICST), Sharif University of Technology, P.O. Box:14588-89694, Tehran, Iran

**Keywords:** EEG, Educational multimedia, Forward encoding models, Temporal response function, Cognitive load, Cognitive neuroscience, Computational neuroscience

## Abstract

Multimedia is extensively used for educational purposes. However, certain types of multimedia lack proper design, which could impose a cognitive load on the user. Therefore, it is essential to predict cognitive load and understand how it impairs brain functioning. Participants watched a version of educational multimedia that applied Mayer’s principles, followed by a version that did not. Meanwhile, their electroencephalography (EEG) was recorded. Subsequently, they participated in a post-test and completed a self-reported cognitive load questionnaire. The audio envelope and word frequency were extracted from the multimedia, and the temporal response functions (TRFs) were obtained using a linear encoding model. We observed that the behavioral data are different between the two groups and the TRFs of the two multimedia versions were different. We saw changes in the amplitude and latencies of both early and late components. In addition, correlations were found between behavioral data and the amplitude and latencies of TRF components. Cognitive load decreased participants’ attention to the multimedia, and semantic processing of words also occurred with a delay and smaller amplitude. Hence, encoding models provide insights into the temporal and spatial mapping of the cognitive load activity, which could help us detect and reduce cognitive load in potential environments such as educational multimedia or simulators for different purposes.

## Introduction

The progress of educational technologies is undeniable; numerous schools now integrate multimedia into classrooms to enhance the learning experience. Making lessons more engaging could help capture learners’ attention effortlessly. However, learners may still encounter difficulties in understanding educational multimedia, which could result in increased cognitive load^1^. Cognitive load (CL) can be defined as a multidimensional load, imposed by cognitive tasks on the cognitive system^2^. The aim of research on CL within the instructional domain is to find methods to reduce it and improve the learning process.

CL has been measured with the use of behavioral data^3^, secondary tasks^4^, eye data^5^ and electroencephalography (EEG)^6,7^. Antonekno^8^ used frequency bands to measure the CL of a reader. Due to the high temporal resolution of EEG, Event-Related Potentials (ERP) studies can also tell us a lot about the temporal dynamics of the brain activity, particularly by repeatedly presenting conditions time-locked to the stimulus onset. The timing of text-picture integration was explored in ERP and ERD/ERS studies^9^. By plotting the time-frequency of the $F_Z$ and $P_Z$ channels, Scharinger concluded that EEG is a valid and practical tool to measure mental processing demand^10^. In another study, DeLeeuw et al.^11^ investigated Mayer’s principle of multimedia learning by measuring ERPs using brief visual distractors. Solis^12^ utilized ERP and secondary tasks to measure CL while participants were driving in a car simulator. Mobile EEG and Brain Computer Interface (BCI) have also been used to measure CL online^13^. Using ERP analysis, Yu^14^ has shown that the degradation of visual stimuli can increase CL. Degraded visual stimuli are intentionally reduced quality or clarity of visual information, such as blurred or pixelated images or text. Sarailoo measured the CL of educational multimedia using machine-learning techniques^15^. For a systematic review, see^16^.

The main issue with the ERP technique lies in its inability to handle complex stimuli, such as those found in educational multimedia. This type of multimedia typically involves the continuous presentation of various elements like pictures, text, animations, speech, and sound. To address this issue and in line with recent studies on the dynamics of brain activity in naturalistic stimuli, we used encoding models. Two of the widely used methods of modeling the brain activity are encoding and decoding^17–20^. Encoding or forward modeling uses stimulus features to predict brain response, while decoding or backward modeling uses brain response to construct stimulus features. Temporal response function (TRF) describes a mapping between some feature of a sensory stimulus and the neural response^21^. TRFs can be used to model the brain activity in different tasks, such as luminance^22^, audio envelope^23^, low or high-order linguistic features^24,25^, second language (L2)^26,27^, music^28,29^, attention^30–33^, and in infants and older adults^34–36^. To the best of our knowledge, no study has investigated the effect of CL on TRFs. To use this encoding model, we employed a continuous stimulus (speech and related visual elements in the form of a series of slides) and recorded a continuous brain response (EEG). Then utilized mTRF toolbox to find TRFs^21,37^. The problem can be addressed using encoding models under two conditions: one with high CL and the other with low CL. Therefore, we analyzed the TRFs of two different educational multimedia to see if the brain responses showed significant differences. For this purpose, we chose two regressors, one for the sensory processing (Audio Envelope), and the other for the higher-order cognition (Word Frequency). The audio envelope describes the changes in sound over time, while word frequency indicates how often a word appears within a corpus. Figure 3a illustrates these regressors.

This study aims to investigate the components of TRF when there is a high CL. The contributions of this work are as follows: (1) Comparing the TRF underlying high vs low CL, and (2) Predicting CL by detecting the relation between components of TRF and behavioral data on the presence of CL. In the section Methods, we describe the experimental design, subjects, and apparatus. Then, in Section “Results”, the TRFs, their performance, and their relation to behavioral data are presented. Finally, in Section “Discussion”, you can see the interpretation of our data, comparison to previous researches, and limitations.

## Methods

### Stimulus

**Figure 1:**
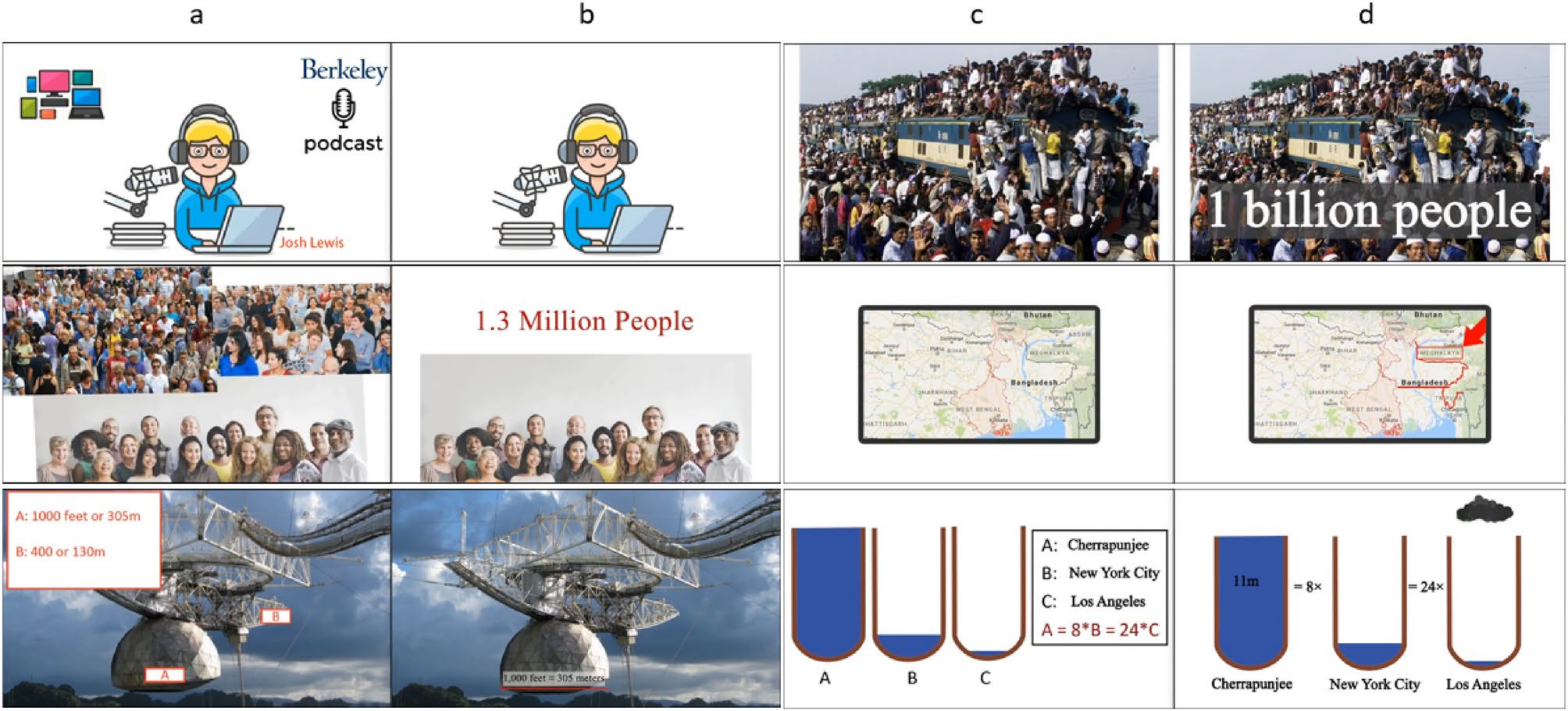
Twelve example frames from designed multimedia. The multimedia is publicly available at https://osf.io/v53np/. (**a**) Three example frames from lesson 11 and without multimedia learning principle (NP) condition. (**b**) The equivalent frames of column (**a**) frames in with multimedia learning principle (P). (**c**) Three example frames from lesson 6 and NP condition. (**d**) The equivalent frames of column (**c**) frames in P condition.

From the two listening files, four educational multimedia files were designed. Each listening file contained two multimedia files with different conditions. We used two different listening files to avoid learning in the second session. These multimedia files were designed according to Mayer’s principles of multimedia learning. The design violated or applied five extraneous principles of multimedia learning, including: (1) Coherence: remove distracting materials. (2) Signaling: highlighting what to focus on. (3) Redundancy: narration and graphics are better than narration, graphics, and text. (4) Spatial Contiguity: relevant text and visuals are physically close together. (5) Temporal Contiguity: corresponding words and visuals are presented at the same time. Find more details about the stimulus in our previous work^15^. Two linguists in English language teaching devised the scenario for making educational multimedia. Then, the multimedia files were created by a motion graphics specialist in the Adobe After Effects CC 2017 v14.2.1.34 software. The two conditions are with principle (P) and without principle (NP). Figure 1 has three example frames from each multimedia. For each condition, there were two lessons: 11 and 6. The duration of lesson 11 is 342 s, and the duration of lesson 6 is 290 s. The listening files are from Oxford’s Open Forum 3^38^; the slides are related images and texts. Each lesson has two versions: P and NP. The multimedia and tasks are presented by software designed for this task, available on GitHub^39^. The audio is not changed in any way.

Both P and NP have the same audio, but they have different slides, which differentiates them. In NP multimedia, we violate the principles according to the rules mentioned by Mayer in Multimedia Learning book^40^. The task has only two sessions, one for each multimedia, and it is not divided by trial. The audio sampling rate is 44100 Hz, and the video is 30 frames per second. The video is played on a CRT display monitor (17 inches; PF790; refresh rate, 75 Hz; viewing distance, 57 cm). To minimize head movements and ensure consistent data collection, the subjects put their head on a chin rest.

### Participants

Thirty-nine university students, aged between 20 and 29 years (mean = 22.8, std = $\pm 2.5$, two females), participated voluntarily after a recruitment announcement. They were randomly assigned to watch lesson 11 NP, then lesson 6 P (n = 21) or lesson 6 NP and then lesson 11 P (n = 18). The data were collected in previous studies^15,41^. Specifically, 29 participants remained in the first condition, and 28 participants remained in the other condition after removing participants due to the following reasons: incomplete recording (n = 2), noisy data (n = 7), to find details see Preprocessing subsection, and too low post-test score (n = 2 in the first condition and n = 1 in the other). All participants reported having normal or corrected-to-normal vision and hearing; None of them had a history of neurological disease.

The first language of all participants is Persian, and their second language is English. They participated in a standard pre-task listening test and accomplished it. In addition, they performed a test similar to the main procedure to familiarize themselves with the main procedure. They signed an informed written consent form before participating in the study. All experimental protocols used in this study were approved by the Iran University of Medical Sciences (IR.IUMS.REC.1397.951). All the methods used in this study were performed in accordance to the guidelines and regulations outlined by the Iran University of Medical Sciences (IR.IUMS.REC.1397.951). The approved protocol was in agreement with the Declaration of Helsinki.

### Data collection protocol

The Participant sits in an adjustable chair in a dim light room while no other sounds disturb them. First, the procedure was explained to them, they saw a one-minute video with different subjects from the main video to become familiar with the environment. The EEG recorded using a 32 channel eWave (https://sciencebeam.com) device with a sampling rate of 1 KHz, and the software is eProbe v6.7.3.0, with a cap of 10–20 system^15,41–43^. Two channels are connected to the mastoid bones used for reference. Visual triggers on the monitor were used to ensure synchronization and, there are two loudspeakers in front of the subject, one on the right and one on the left.

**Figure 2:**
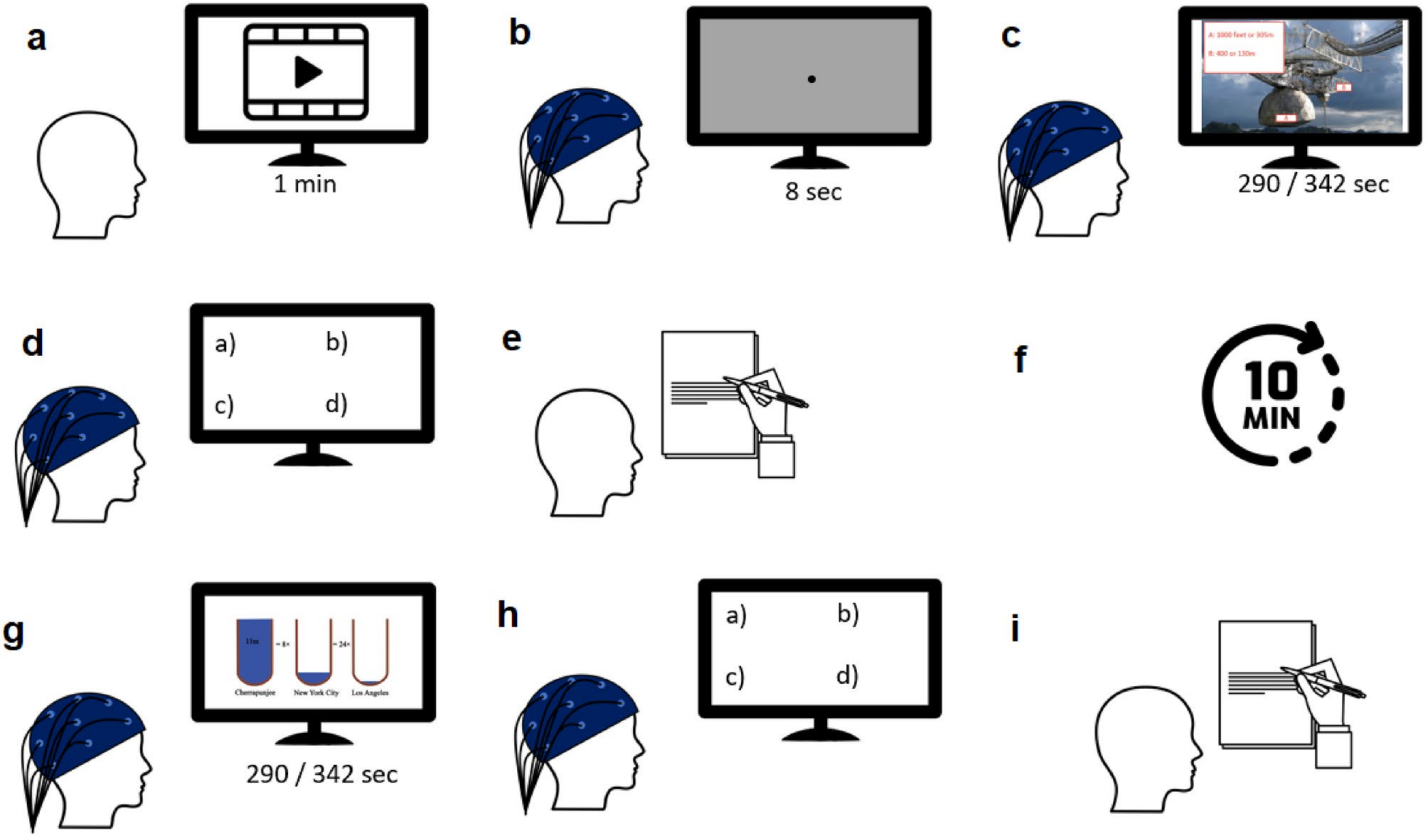
Task Paradigm. Two groups were formed; in one group, Lesson 11 was played first, followed by Lesson 6. In the other group, the sequence was reversed. At the beginning of the experiment, the participants were randomly assigned into one of two groups. (**a**) A one-minute video was shown to the participants to familiarize them with the main experiment. (**b**) Participants were fixated on a point on a gray screen for 8 s to record their baseline EEG. (**c**) While recording their EEG, participants watched Lesson 6 or 11 under the NP condition. (**d**) They attend in a four-answer post-test. (**e**) Participants completed a pen-and-paper NASA-TLX questionnaire. (**f**) They took a ten-minute rest. (**g**) While recording their EEG, participants watched Lesson 6 or 11 under the P condition. (**h**) Again, they engaged in a four-answer post-test based on the most recent multimedia lesson they viewed. (**i**) Finally, they completed another NASA-TLX questionnaire for their last multimedia.

As mentioned in the Stimulus section, there are four multimedia, and subjects are randomly assigned to two groups. One group saw Lesson 11 NP and Lesson 6 P, while the other viewed Lesson 6 NP and Lesson 11 P. The experiment consisted of two sessions: first, the eye tracker was calibrated (the eye data is not analyzed in this study, which just served to ensure that subjects were attending to the monitor). Following a countdown, the multimedia presentation began. Then, they are supposed to answer the questions to assess how much they had learned and whether they had paid attention. Subsequently, they completed a paper-based NASA-TLX questionnaire in their first language^44,45^. After a short break, the second session started with the same procedure but with other multimedia and conditions. The entire procedure is illustrated in Fig. 2. Find more details in the Materials and Methods section of our previous work^15^.

### Analysis

EEG data preprocessing and modeling were performed in MATLAB (MathWorks Inc.), EEGLAB^46^, multivariate temporal response function (mTRF) toolbox^21^, and modified encoding scripts from the CNSP 2021 workshop (cnspworkshop.net).

#### Preprocessing

The following steps were undertaken for data preprocessing: First, we filtered the data using both a 1 Hz high-pass filter and a 10 Hz low-pass filter, since neural representation for speech is suggested to occur within this frequency range^47^. To remove noise, we first epoch data to 1 s, then a 200 ms sliding window moved along the EEG data, and whenever the standard deviation of a window exceeds 100, we removed that epoch by replacing it with zero^34^. If a channel has more than 100s, we interpolated that channel, and if a subject has less than 100 s of clear data remaining, that subject is removed from the rest of the analysis. Then we run the Independent Component Analysis (ICA), and to remove non-neural components, we use ICLabel^48^. Then downsampled the data to 250 samples per second and divided it into 10 trials.

#### Extracting of features from multimedia

**Audio Envelope** The audio was extracted from the multimedia. To prevent the aliasing effect, we first applied a 1 Hz high-pass filter and a 10 Hz low-pass filter, then downsampled the audio to 250 Hz, similar to the EEG data. Finally, we pick the absolute value of the Hilbert transform as the audio envelope regressor^49^.

**Figure 3:**
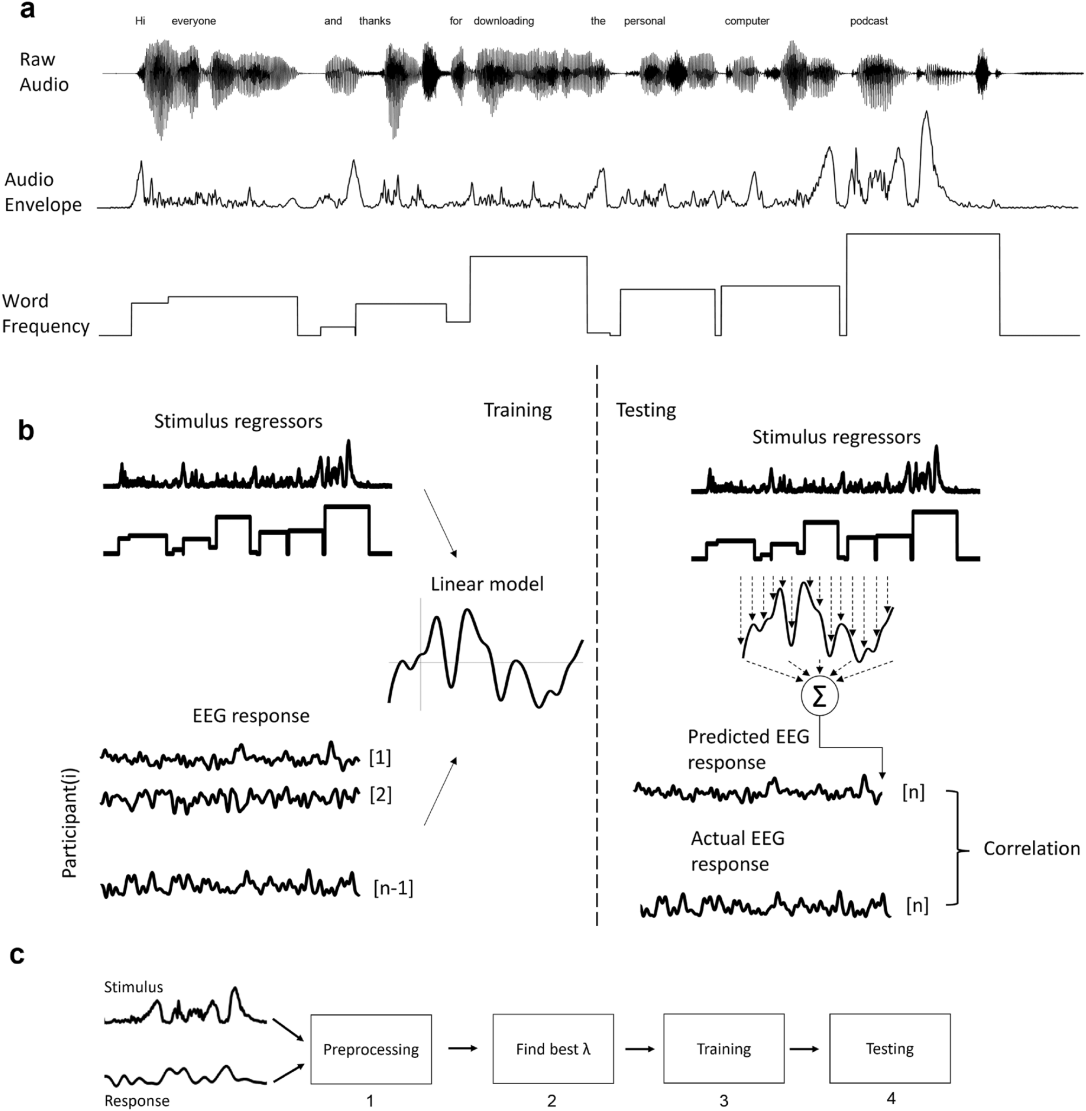
Modeling procedure. (**a**) Regressors extracted from the multimedia are audio envelope and word frequency. (**b**) Generic modeling of continuous EEG data, including the training and testing phase. In training, we find our TRF by optimizing the error of the linear model with n-1 participants and our regressors. Then in the testing phase, predicted response is the convolution of regressors with TRF. Correlation is calculated between actual and predicted EEG responses. (**c**) Procedure of data analysis. (1) Both stimulus and response (EEG) are preprocessed as described in Preprocessing and Extracting of features from multimedia subsection. (2) To find the TRF, first we need to optimize our ridge regression model by finding the best lambda. With a logarithmic vector of lambda values and Eq. (2) we find TRFs for different lambda. Then by Eq. (1), we predict the response for each $\lambda$ and then calculate the correlation between the predicted and true EEG response. Finally we choose the $\lambda$ of the highest correlation for the training step. (3) We split data into train and test. In the individual models $80\%$ of each subject and in Generic models n-1 subject. With the best $\lambda$ found in the previous step and Eq. (2) we find TRF weights. (4) With the test data and founded TRF in the previous step, we calculate the predicted response with Eq. (1). Then, we calculate the correlation of true and predicted EEG response.

**Word frequency** To compute the word frequency regressor, we manually identified the exact timings of the start and end of each word using Praat^50^. Subsequently, we used the SUBTLEX database^51^ to determine the logarithmic value of the word frequency. Here, *wf* stands for the frequency of occurrence of that word in the database, which is calculated based on the subtitles of English movies and TV series. As the highest value in the dataset is 6.329, we set 6.33$-\log _{10}wf$ for the duration of each word. In this way, more frequent words are assigned lower values, infrequent words are assigned higher values, and for periods when we do not have a word, we set the value to zero^52^. These features are illustrated in Fig. 3a.

#### Temporal response function

To model the relationship between the stimulus features and neural data, we use a linear model, specifically ridge regression^53^. The model predicts neural data by a set of time-lagged weights convoluted by input; here is the stimulus feature^22^.

1 $$\begin{aligned} R(t) = \sum _{1}^{\tau } TRF(\tau )S(t - \tau ) + \epsilon (t) \end{aligned}$$

In Eq. (1), R is the response at a specific channel at time t, TRF is the temporal response function or model weights, S is the matrix of time-lagged input feature or in other words, design matrix and $\epsilon$ is noise or variation that cannot be explained by the model.

To determine the TRF, we can solve an optimization problem by attempting to minimize the error between the predicted and actual responses. If we do so, then the solution is:

2 $$\begin{aligned} TRF = \frac{S^TR}{S^TS+\lambda I} \end{aligned}$$

In Eq. (2) I is the identity matrix and lambda is a constant value found in the optimization procedure. For the implementation, we use the mTRF Toolbox^21^ in MATLAB. First, we divide the continuous data into ten trials. For the $\tau$ parameter, we set it between $T_{min} = -200$ ms and $T_{max} = 1000$ ms^34^, as numerous previous studies have shown that the processing of auditory information is done in the brain in less than 1 s. Figure 3b shows the modeling procedure.

#### Choosing the optimal regularization parameter $\lambda$

As mentioned in the section Temporal response function, we first equally divide the data into ten trials, then run cross-validation with 80% of the data and use the remaining 20% for testing, ensuring that the model never sees this portion of the data during the training phase. For the training data, we apply the leave-one-out cross-validation method with a range of lambda values to identify the optimal one^54^. For each lambda value, we predict the neural response, calculate the correlation between the predicted and actual data, and select the highest correlation value.

As with EEG data, there are many features and few samples; usually, overfitting is inevitable. To solve this issue, ridge regression uses a penalty parameter named $\lambda$. To optimize the model, or in other words, to find the best value for lambda, we create twenty-one lambdas from $10^{-5}$ to $10^5$ with a 0.5-step size in power^47^.

#### Evaluation of temporal response function

After finding the best value for lambda, we train the model to determine the TRF weights and then test the model on the held-out data. We use correlation to measure how good the prediction of neural response is^31^. As the correlation values are small, we must be sure that they are meaningful. To achieve this, we use a shifted control method.

We create another set of data while the response is circularly shifted by 2 s to ensure that there is no relation between the input stimulus feature and the output neural response. Then, we apply the same modeling process and calculate the model’s performance by measuring the correlations^34^.

## Results

Participants watched two multimedia, P and NP, while their EEG and eye data were recorded. Here, we use only the EEG data in our analysis. After that, to be sure that they attended to multimedia and assess their learning, they participated in the performance test. We also gave them a NASA-TLX questionnaire to ensure that the two versions of the multimedia presentations indeed differ in the levels of CL imposed.

### NASA-TLX and post test results

**Figure 4:**
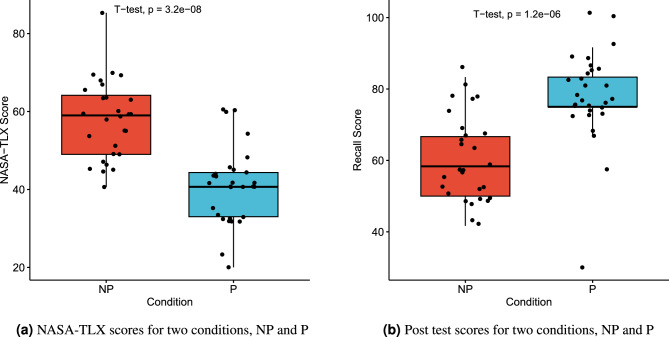
Results of the NASA-TLX and post tests. The scores are in the range of 0 to 100. Each dot represents a subject. The p-value of the t-test is annotated in the figure. (**a**) The results for NASA-TLX task load index and (**b**) The results for our post test.

To evaluate whether our designed multimedia in the P and NP conditions affected the subject’s CL and check if they were attentive to the multimedia presentations, we asked subjects after watching multimedia to answer 12, four-option questions from multimedia, and then filled out the NASA-TLX questionnaire. We expected to see a significant difference between the P and NP conditions. The results are shown in Fig. 4. As shown, participants who watched the P multimedia reported a lower overall CL compared to those who watched the NP multimedia, and the difference is significant (t(55) = 6.4407403, p = 3.2E−08). The statistical result for post-test also indicates that the NP multimedia significantly increases CL, and as a result, the post test score for the P condition is higher than that for the NP condition (t(55) = 5.439136037, p = 1.2E−06).

### Temporal response function

**Figure 5:**
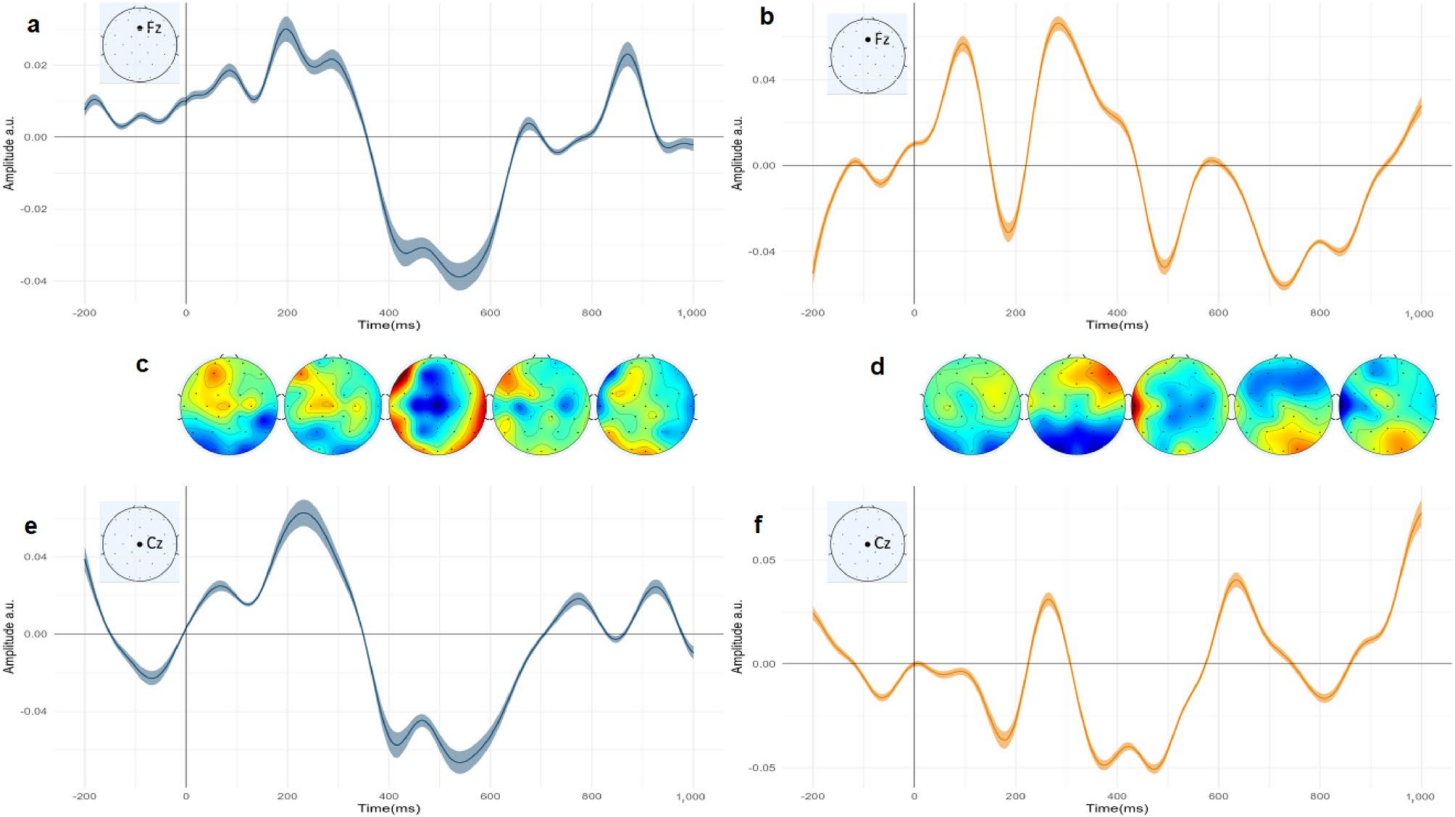
Compare Audio envelope temporal response functions of P and NP condition. Standard Error of the Mean (SEM) and 95% confidence interval, as well as topographies of related brain activity over time. The light blue TRF represents the P condition, while the orange one corresponds to the NP group. The corresponding location on the electrode map is illustrated in the corner of each TRF (**a**) Audio envelope (AE) TRF for channel Fz, for P condition. (**b**) AE TRF for channel Fz, for the NP condition. (**c**) Topographies of AE for the P condition. Each topoplot is responsible for the corresponding time in the x-axis of TRF. For example, the first one is from 0 to 200 ms. (**d**) The same topographies for the NP condition. (**e**) AE TRF plotted for channel Cz for the P condition (**f**) AE TRF for the NP condition, channel Cz.

To see the effect of neural signature in two groups, we try to fit two different regressors, both in the individual and the generic manner in which the model is trained by n-1 subjects and tested by the remaining one (leave-one-out method)^34^. Our regressors were the Audio Envelope and Word frequency. Interestingly, we observed robust, consistent, and different activities in both groups. In the NP group, we saw both early and late neural components, a similar pattern to Jessen et al.^34^, and in the P group, as illustrated in Fig. 5, we see two major late components and small early components. In the NP group (channel Fz), the components occur at 100, 200, 300, 500, and 750 ms. In the P group, we observe the components occurring at 400 and 700 ms (n = 29). These TRFs are calculated and plotted for channels Fz and Cz. The average response is shown with a line and the standard error of the mean SEM ($95\%$ confidence interval plotted with shadow around the mean). We also plot topographies of brain activities for each 200 ms and put them in a corresponding place on the x-axis. For the P group, we see that around 400 to 600 ms a high activity is happening in the central and frontal regions, with mostly left-lateralized activity. In the NP condition, the duration of activity at the same time intervals was shorter than in the P condition.

**Figure 6:**
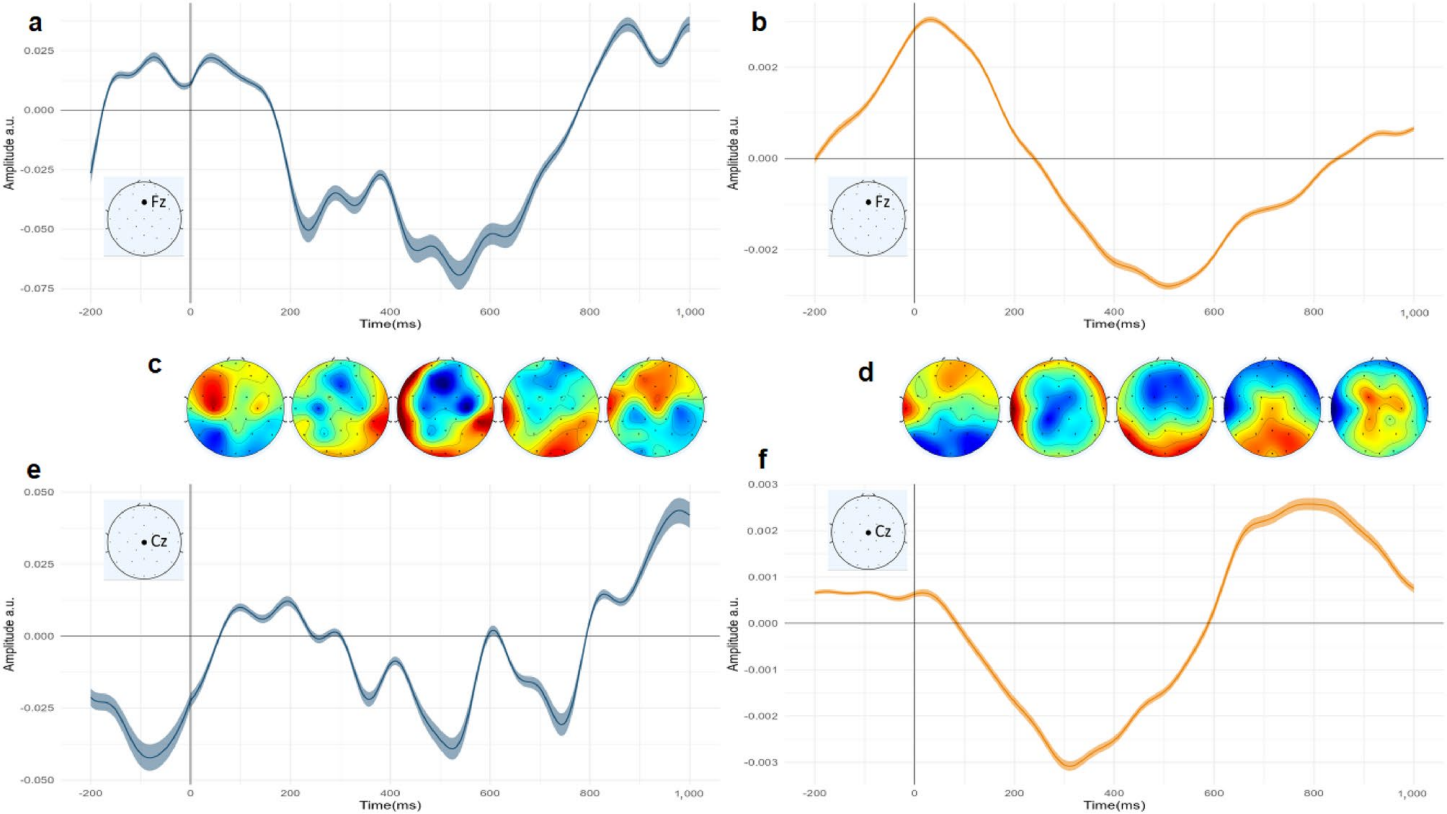
Compare Word Frequency TRFs of P and NP condition. SEM and 95% confidence interval. And topographies of related brain activity across time. The light blue TRF is for P and, the orange one is for the NP group. The corresponding location in the electrode map is illustrated in the corner of each TRF. (**a**) Word frequency (WF) TRF for channel Fz, for P condition. (**b**) WF TRF for channel Fz, for NP. (**c**) Topographies of WF for P condition. Each topoplot is responsible for the corresponding time in the x-axis of TRF. For example, the first one is from 0 to 200 ms. (**d**) Same topographies for the NP condition. (**e**) WF TRF plotted for channel Cz, condition P. (**f**) WF TRF for condition NP, channel Cz.

For the word frequency regressor, we also observed a robust and different pattern between the two groups. In the P group (channel Fz), Fig. 6 shows three main components occurring at 100, 500, and 850 ms, while in the NP group, we see two main smoother and lower activities. Interestingly, the amplitude in the NP condition was smaller than that in the P condition. The topographies indicate that, similar to the audio envelope regressor, there is a high activity from 400 to 600 ms, but it is happening in frontal electrodes rather than centro frontal electrodes in the auditory P condition. For the NP condition, the activity that is happening is not as strong as that in the P condition.

### Generic versus individual response functions

There are two approaches for modeling our data: individual and generic models. In individual models, we train and test our model with one specific subject, whereas in generic models, we train it with n-1 subjects and test it with the remaining subject. When dealing with limited data, generic models are recommended.

**Figure 7:**
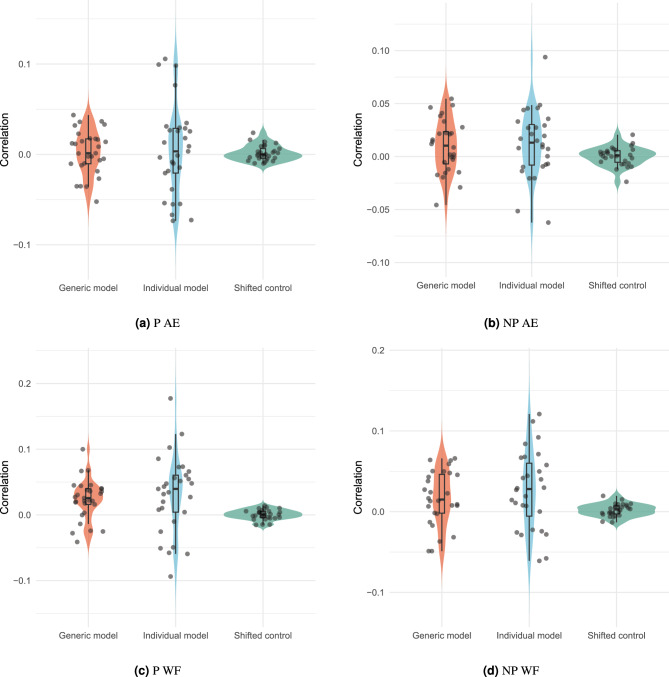
Predictive accuracy of Word Frequency and Audio Envelope for P and NP conditions.The correlation between predicted and actual EEG on 20$\%$ of data for two models. Generic (leave-one-out) and Individual models, We also plot the performance for the shifted control condition. All for channel Fz. (**a**) Performance for audio envelope TRF for P condition (**b**) Performance for audio envelope TRF for NP condition (**c**) Performance for Word Frequency TRF for P condition (**d**) Performance for Word Frequency TRF for NP condition.

In the previous section, we investigated TRFs in a generic manner. Here, we estimate the predictive accuracy of neural data in our forward model. Figure 7 shows the results for both the NP and P groups. As the correlation values are small, we must be sure that they are not just random values, so we shift the EEG signals by 2 s from our regressor signal. Two seconds could be sufficient to ensure that the neural signal is no longer related to the stimulus anymore^55^. Then we generate shifted trials.

First, we see that the mean of the correlations is greater than zero, and they are not close to zero, as in the shifted trials. As expected, individual performance has more variance than generic models. In addition, we observe that the performance for word frequency is higher than that of the audio envelope.

### Relation between features of components and behavioral data

**Figure 8:**
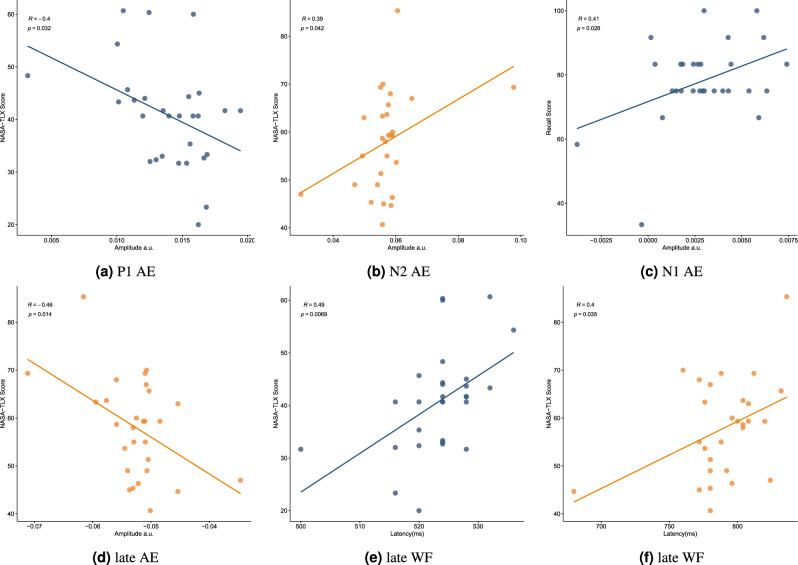
Relation between features of components and behavioral data The relation found for audio envelope (AE) and word frequency (WF) in two conditions P and NP, for electrode Cz and Pz while our behavioral data is NASA-TLX and post test. Each circle represents a subject. The blue color is for the P condition, and the orange is for the NP condition. (**a**) Amplitude of the first component in AE P to NASA-TLX, channel Fz (**b**) Amplitude of the second component in AE NP to post test, channel Fz (**c**) Amplitude of the first component in AE P to NASA-TLX, channel Cz (**d**) Amplitude of late component in AE NP to NASA-TLX, channel Cz (**e**) Latency of late component in WF P to NASA-TLX, channel Cz (**f**) Latency of late component in WF NP to NASA-TLX, channel Cz.

To see if there is any relation between generic TRF components and behavioral data, we divide the time range of TRF into four parts. First from 0 to 150 ms (N/P1), second from 150 to 250 ms (N/P2), third from 250 to 350 ms (N/P3), and the late component for weights found greater than 350 ms (late positivity/negativity). Then, we extract the amplitudes and latencies of each component in the TRFs for the audio envelope and word frequency in the two conditions, NP and P, both in the Cz and Fz channels. We calculate Pearson’s correlation and the corresponding p-value with behavioral data, including both the subject’s performance on post test and NASA-TLX scores. The results are presented in Table 1 and Fig. 8.

**Table 1:** Correlation between TRF features and behavioral data. The maximum and minimum amplitude of components and the latency of them found for each subject. Then, correlations and p-values between TRF features and the subject’s post-test score and NASA-TLX index were calculated and reported. The results show that there is a relation between behavioral data and TRF features.

Component	Feature	Ch	Stimulus	Behavioral measure	Condition	r	p values
P1	amp	Fz	Audio Envelope	NASA-TLX	P	-0.400080395	0.031518771
N2	amp	Fz	Audio Envelope	NASA-TLX	NP	0.385990373	0.042484903
N1	amp	Cz	Audio Envelope	post test	P	0.413893684	0.025614097
Late Negativity	amp	Cz	Audio Envelope	NASA-TLX	NP	-0.461061067	0.013535079
Late Negativity	lat	Cz	Word Frequency	NASA-TLX	P	0.490181391	0.006946695
Late Positivity	lat	Cz	Word Frequency	NASA-TLX	NP	0.400161124	0.034859431

## Discussion

In this study, we presented two types of multimedia to participants, P and NP, while simultaneously recording their EEG. The multimedia presentations had the same audio, but the slides were designed in a way to impose high or low CL. We observed that while audio and words had the same content in the two conditions, the brain processed them slightly differently. In addition, we found relationships between the behavioral data and our forward encoding model features. This may help us understand the neural mechanisms underlying high CL. Detecting high CL in ecologically valid setups may help avoid potential losses. It can be beneficial in the following fields: Human-Computer Interaction (HCI), User Experience (UX) Design, Educational technology, Automotive, Aerospace, Industrial engineering, Designing workspaces, and Medical Equipment and Healthcare. Attaining a deeper understanding of the CL would help engineers design systems and environments which use cognitive resources optimally, leading to an enhanced performance, safety, and well-being.

CL can be assessed subjectively, by performance tests and physiological data^56^. Questionnaires like NASA-TLX and performance tests are slow, as their indexes are only available at the end of the task. Thus, for a more comprehensive evaluation of CL over time, we can turn to physiological data such as EEG signals. Previous research indicates that ERP is a suitable method for demonstrating changes in the load on working memory and the various components influenced by different tasks^57^. While ERP provides a high time resolution, TRF enables us to utilize more naturalistic and intricate stimuli commonly encountered in real-world situations. In addition, TRF analysis is effective when we need more engaging stimuli; and so it would be helpful in special groups of subjects such as children and patients.

The early auditory evoked potentials (AEP) are N1 and P2. Several studies show the relation between N1 amplitude and latency with attention. There is a positive correlation between N1 amplitude and attention, that is, the higher the amplitude of N1, the more attention exists^58–60^. Analogous to N1, P2 is modulated by attention, in contrast to N1, it has a negative correlation with attention, in other words, the larger the amplitude of P2 the lower the attention is^61^. Also, it has been shown that P2 amplitude increases during sleep^62^. We find a P1 relation to the NASA-TLX score, which supports the literature^12^ indicating that P1 amplitude has a negative correlation with CL. A high load may occur because of Mayer’s rules that have been violated, such as extra materials on the slide, the distance between learning objects in the slide, and unsynced audio, all of which can cause a lack of attention. We also observe that our N1 component has a positive correlation with the post-test score. Previous studies have shown that N1 is responsible for speech segmentation. Detecting the starting point of a word is important for speech comprehension. The earlier and the larger N1, the higher the speech comprehension^63–65^. Ihara et al. also saw that the higher the proficiency in L2, the higher the N1 amplitude^27^. Marcos et al. also see in an ERP study that drivers with a high mental workload show lower N1 amplitude^12^. Previous ERP studies have shown that early components, such as N1 and P2, in audiovisual tasks occur earlier and with lower amplitude^66–68^. This may explain why we see that early components of our TRFs are smaller, and their latency is shorter.

In late components, there is an N400 component. Several studies have shown that N400 is responsible for semantic processing^69,70^. The N400 appears with different modalities, such as audio, words, or pictures. It is suggested that two cognitive processes occur during the N400: first, accessing semantic terms from long-term memory; and second, integrating semantic information together. The amplitude of our late components has a negative correlation with the NASA-TLX score, and the latency of the late components has a positive correlation with the NASA-TLX score. Newman et al. also saw a negative correlation between N400 amplitude and speech comprehension score; we can say that as CL increases, there would be lower space for semantic processing^71^ . We also see that there is a positive correlation between the late component latency and the NASA-TLX score. Ihara et al. demonstrated that Japanese individuals with higher L2 proficiency have an earlier N400, indicating that the brain processes the meanings of words earlier than in those with lower proficiencies^27^. Our N400 may occur later under high CL because more time is needed to process semantic information in NP condition.

Figure 8 and Table 1 suggest that CL and learning in educational multimedia can be predicted using merely a small portion of EEG recording, by analyzing the amplitude and latencies of TRF components of only one channel. Friedman et al.^72^ also predicted CL in Raven’s 2 IQ test, where participants had to solve 36 questions without a time limit while their EEG was recorded. Other studies have used machine learning and statistical methods to measure CL using the same dataset as our research. However, they have not addressed cognitive processes such as audio or linguistic^15,41^. For a review on the CL recognition with EEG see^73^.

This study has some potential limitations. Firstly, our focus was on second-language learners. However, to accurately differentiate neural activities related to CL, it would be more appropriate to utilize stimuli in the participants’ native language. For instance, even though presenting an excessive amount of text may contradict Mayer’s principle, it can assist second-language learners in better understanding multimedia content. Secondly, the hypothesis that every moment in the NP multimedia lacks principles or that each moment in the P multimedia follows principles is probably not correct. For example, in certain moments of NP multimedia, there is both speech and image, these instances could also meet the criteria for the P condition. In addition, by using eye tracking alongside EEG recording, one could also detect where subjects focus their attention. This will allow us to extract a more accurate regressor from the stimuli to use in the forward model^74,75^. Finally, given advancements in technology, particularly in virtual reality (VR) and augmented reality (AR), we suggest examining the effects of these tools on CL^76^.

In conclusion, in this study we developed a model to predict CL using EEG signals recorded while participants viewed educational multimedia. The findings indicate that CL influenced the early and N400 components of TRF. Our results highlight the potential to assess CL and learning using TRFs, eliminating the need for traditional paper questionnaires.

## Data Availability

The raw EEG data and multimedia for TRF analysis are available at https://osf.io/v53np/.
